# Clinician Distress: A Concept Clarification

**DOI:** 10.1093/geroni/igaa057.783

**Published:** 2020-12-16

**Authors:** Anessa Foxwell

**Affiliations:** University of Pennsylvania, Philadelphia, Pennsylvania, United States

## Abstract

In 2017, The National Academy of Medicine created the Action Collaborative on Clinician Well-Being and Resilience given the staggering statistics around distress and burnout, which is particularly high while caring for seriously ill older adults. This distress may be physical, emotional, spiritual, moral, ethical, existential or in degrees and combinations of multiple forms. Many crossover concepts are present in the literature, including moral distress, role stress, compassion fatigue, empathy, emotional labor, post-traumatic stress disorder, vicarious traumatization, and second victim. Because of the number of crossover concepts, we employed the Norris concept clarification method examining similar concepts to create an operational definition, construct a model, and develop possible research hypotheses of clinician distress. Articles were identified through PubMed, CINAHL, PsyhINFO, and SCOPUS with the expert assistance of a nurse librarian. Similar concepts in the literature were compared and contrasted with an emphasis on what encompasses clinician distress. Based on synthesis of the literature and existing concepts, clinician distress is defined as experiencing one or more negative emotions before, during, or after a clinical encounter that impacts the quality of care the clinician is able to provide to the patient. The result of this analysis provides some clarity around clinician distress and its surrogate terms, which presents opportunities for further investigation.

